# Unilateral biportal endoscopic debridement with antibiotic-loaded cement beads implantation for lumbar spinal infection: a preliminary report on feasibility and early clinical outcomes

**DOI:** 10.3389/fcimb.2026.1752848

**Published:** 2026-03-18

**Authors:** Gao Ze, Zhenbin Cai, Yiwen Xu, Yuxuan Wu, Zijian Cheng, Yiyi Chen, Zhuolong Xiong, Jing Wang

**Affiliations:** The First Affiliated Hospital of Jinan University, Guangzhou, China

**Keywords:** antibiotic-loaded cement beads, clinical efficacy, lumbar spine, minimally invasive surgery, spinal infection, unilateral biportal endoscopy

## Abstract

**Objective:**

A preliminary evaluate was conducted on the feasibility and clinical efficacy of unilateral biportal endoscopic (UBE) debridement combined with intervertebral antibiotic-loaded cement beads (ALCBs) implantation for lumbar spinal infection.

**Methods:**

We retrospectively analyzed 12 patients with lumbar spinal infections who underwent the procedure between March 2021 and June 2024. Surgical details and complications were documented. Clinical outcomes were evaluated using VAS for pain, ODI, ASIA impairment scale, inflammatory markers, and radiographic parameters, which were measured at multiple timepoints before and after surgery.

**Results:**

All 12 patients successfully underwent the procedure. The mean operative time was 85.58 ± 13.15 minutes, with an estimated blood loss of 51.83 ± 11.50 mL and a mean hospital stay of 6.50 ± 1.45 days. The average follow-up period was 16.50 ± 4.20 months. Significant improvements were observed in all clinical and laboratory outcomes (all overall P< 0.05). Back pain VAS decreased from 6.50 ± 1.17 preoperatively to 2.50 ± 1.72 at final follow-up; ODI improved from 67.42 ± 2.27 to 19.17 ± 3.76. Inflammatory markers also showed significant reductions: WBC (13.47 ± 4.12 to 8.17 ± 0.85 ×10^9^/L), ESR (59.33 ± 16.04 to 12.33 ± 4.42 mm/h), CRP (71.63 ± 25.56 to 3.42 ± 1.86 mg/L), and PCT (1.59 ± 1.06 to 0.04 ± 0.02 ng/ml). ASIA grades improved significantly (preoperative vs. final follow-up, P< 0.05). Radiographically, intervertebral height was maintained (IHI from 0.19 ± 0.04 to 0.23 ± 0.02), and segmental range of motion decreased from 8.02 ± 1.46° to 2.85 ± 1.10° (P< 0.05). 75% of patients achieved spontaneous anterior bony fusion at final follow-up. No complications such as cauda equina injury, cerebrospinal fluid leakage, or pars interarticularis fracture occurred. The clinical success rate was 83%.

**Conclusion:**

This preliminary study suggests that for carefully selected patients with lumbar spinal infections, the combined UBE debridement and ALCB implantation technique may represent a feasible and promising minimally invasive option, offering thorough debridement, sustained local antibiotic delivery, and mechanical support. These initial findings, based on a small single-center series, warrant validation through prospective comparative trials.

## 1 Introduction

Spinal infection represents a significant challenge in spinal surgery, potentially leading to progressive vertebral destruction, spinal instability, intractable pain, and neurological deficits due to nerve compression, substantially impairing patients’ quality of life (Zou et al., 2025). The epidemiology of spinal infections exhibits a bimodal age distribution, with higher incidence rates among in young children and the elderly (Issa et al., 2018). Recent studies indicate that male patients account for 58.2% of cases, corresponding to a male-to-female ratio of 1.5. The lumbar spine is the most commonly affected region (60.3% of cases), followed by the thoracic spine (31.6%), cervical spine (14.7%), and sacrum (0.1%) (Lang et al., 2023). Early-diagnosed infections can be successfully treated with conservative medical management. However, surgical intervention becomes necessary in cases of persistent neurological deterioration or spinal instability (Tsantes et al., 2020; Di Rienzo et al., 2025). Traditional open surgery requires extensive muscle stripping and laminectomy, which is highly invasive and can compromise spinal stability. Furthermore, it often necessitates complex instrumented reconstruction, increasing surgical risks and patient burden (Connor et al., 2013; Choi et al., 2020).

Recent advancements in minimally invasive concepts and techniques have revolutionized the management of spinal infections (Verdú-López et al., 2017; Ishii et al., 2022). Unilateral biportal endoscopic (UBE) technology, which features independent visual and operational channels, significantly improves visualization of the surgical field and facilitates operative manipulation. It is now widely used in the treatment of complex spinal infections, allowing for minimally invasive neural decompression, precise debridement of lesions, and endoscopic fusion (Wang et al., 2023; Chu et al., 2025). Concurrently, local antibiotic delivery strategies offer notable clinical benefits. Antibiotic-loaded bone cement serves as an effective carrier that releases high concentrations of antibiotics directly at the infection site in a sustained manner. This approach significantly elevates local drug levels, combats biofilm-forming bacteria more effectively, and reduces the systemic toxicity commonly associated with high-dose intravenous antibiotics (Kühn et al., 2017; Berberich et al., 2023). Furthermore, its mechanical support upon solidification helps reduce reliance on internal fixation (Gramlich et al., 2023).

This study presents an innovative combination of UBE minimally invasive technology and local antibiotic delivery using antibiotic-loaded bone cement. ALCBs are precisely implanted into the intervertebral space during UBE-assisted debridement, enabling thorough removal of infected tissue and highly efficient local antibiotic delivery through a minimally invasive approach. We hypothesized that this combined technique would effectively control infection, improve clinical outcomes, and maintain spinal stability while minimizing surgical trauma. To preliminarily evaluate this hypothesis, we retrospectively analyzed the clinical data of 12 patients with lumbar spinal infections treated with this approach, aiming to assess its feasibility and early efficacy as a potential minimally invasive strategy for managing this condition.

## 2 Materials and methods

### 2.1 General information

This retrospective case study reviewed the clinical data of 12 patients with lumbar spinal infections who met the inclusion and exclusion criteria and were treated at our institution between March 2021 and June 2024. Given the exploratory nature of this novel technique and the relatively low incidence of the condition, and as this retrospective study aimed to include all consecutive eligible patients, a formal prospective sample size calculation was not performed. The cohort consisted of 7 males and 5 females, with a mean age of 64.33 ± 5.10 years (56-73 years). All patients underwent a single-stage posterior approach surgery involving UBE-assisted debridement and intervertebral implantation of ALCBs. Preoperative assessments included a detailed neurological examination, anteroposterior and lateral lumbar radiographs to evaluate vertebral and intervertebral space alterations, 3D reconstructed lumbar CT scans to assess bone destruction in the vertebrae and appendages, and lumbar MRI to examine bone and soft tissue lesions as well as spinal nerve involvement.

For patients with pyogenic spondylitis, the medication administered intraoperatively was Vancomycin Hydrochloride (brand name: Vancocin; active ingredient: vancomycin hydrochloride, 0.5 g [500,000 units] per vial; Eli Lilly and Company, USA; National Drug Approval No.: HJ20140174). For patients with spinal tuberculosis or Brucella spondylitis, the medication administered was Streptomycin Sulfate (brand name: Meiluo; active ingredient: streptomycin sulfate, 1g [1,000,000 units] per vial; Dalian Meiluo Pharmaceutical Factory, China; National Drug Approval No.: H21021674). The bone cement used was Polymethylmethacrylate (PMMA) bone cement (brand name: Refobacin^®^ Bone Cement R; active ingredient: gentamicin, 40 g per bag; BIOMET France; Product Registration No.: 3003940001-3).

This study received approval from our hospital’s Ethics Committee (KY-2025-071). All patients provided written informed consent prior to surgery.

### 2.2 Inclusion and exclusion criteria

Inclusion criteria: (1) Definitive diagnosis of lumbar spinal infection with no improvement after at least 4 weeks of culture-guided intravenous antibiotic therapy, followed by clinical and inflammatory marker reassessment; (2) Presence of complications such as vertebral destruction or nerve compression; (3) Pre-existing comorbidities that were under control through systematic treatment.

Exclusion criteria: (1) Concomitant spinal pathologies, including severe thoracolumbar degenerative disease, significant scoliosis or kyphosis, adjacent vertebral fracture, or spinal tumor; (2) Known allergy to Gentamicin or Vancomycin; (3) Advanced age, poor general condition, or severe systemic comorbidities (e.g., cardiopulmonary dysfunction) rendering the patient unfit for surgery; (4) Incomplete medical records or loss to follow-up.

### 2.3 Surgical technique

**Figure 1:**
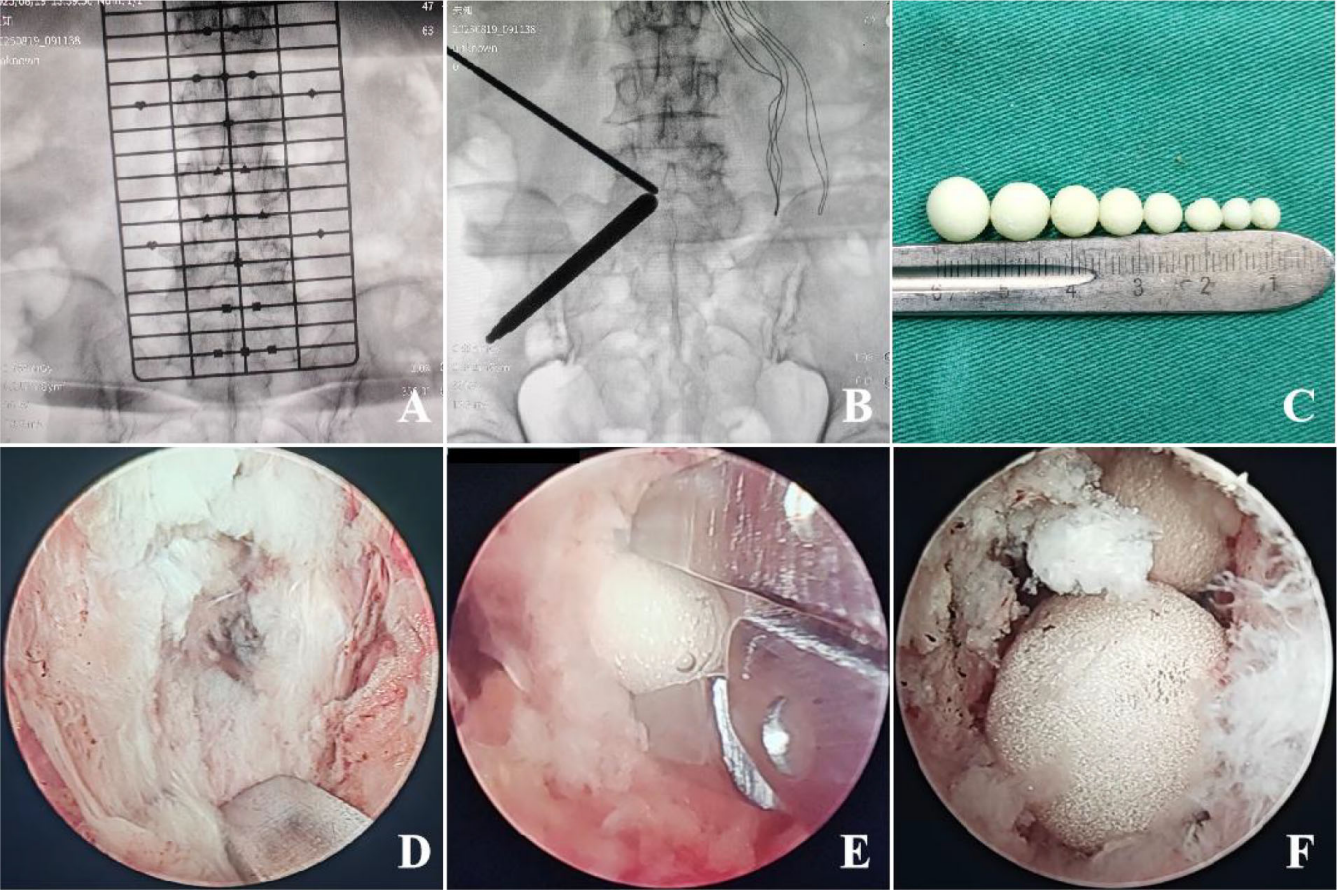
Intraoperative photographs of representative cases: **(A)** Preoperative fluoroscopy confirms the surgical level and incision sites. **(B)** Intraoperative fluoroscopy guides the establishment of the UBE corridor and determines the landing point. **(C)** Antibiotic-loaded bone cement beads are prepared in various sizes. **(D)** Debridement of necrotic disc material and purulent tissue from the intervertebral space. **(E)** A pituitary rongeur is used to insert antibiotic cement beads into the disc space. **(F)** A final larger cement bead is placed to seal the intervertebral space.

All procedures were performed by the same experienced spine surgeon using the UBE technique. General anesthesia was administered with the patient in a prone position and the abdomen left free. The target surgical level was localized and the incision sites were marked under C-arm fluoroscope guidance. After routine disinfection and draping of the surgical site, a sterile adhesive film was applied, followed by the establishment of two portals on the patient’s back, which served as the viewing and working portals, respectively. Under endoscopic guidance, the paraspinal muscles were minimally stripped to expose the superior and inferior laminae. A drill was used to trim the free edge of the lamina clockwise toward the superior and inferior attachment points of the ligamentum flavum until the bilateral ligamentum flavum could be detached and completely removed. The dural sac and nerve roots were then adequately exposed, and a nerve root retractor was used to gently retract them contralaterally. Upon accessing the infected lesion, granulation tissue or other suspected infected tissue was collected and sent for pathogenic microbial next-generation sequencing (NGS) and bacterial culture. Necrotic disc material, pus within the spinal canal, and debris in the intervertebral space were thoroughly debrided and copiously irrigated with normal saline. One gram of vancomycin powder or 1g of streptomycin powder were mixed into 40g of PMMA bone cement and shaped into cement beads of varying diameters (3-10mm). A pituitary rongeur was used to sequentially implant the ALCBs into the intervertebral space. A final larger bead was placed to seal the opening and prevent migration of the beads into the spinal canal. A nerve dissector was carefully used to verify that no cement bead contacted the dural sac or nerve roots, thereby ensuring neural safety (**Figure 1**). Intraoperative C-arm fluoroscopy confirmed satisfactory filling and positioning of the cement beads. A water-stop test was performed to rule out active bleeding. After removal of the working portals, a drainage tube was placed, and the incision was closed routinely.

### 2.4 Postoperative management

Postoperative sensitive antibiotic therapy was promptly adjusted based on NGS and bacterial culture results. A multidisciplinary team dynamically tailored the regimen: intravenous therapy was guided by clinical response and trends in inflammatory markers, followed by an oral course to complete a total duration of 4–6 weeks, with final length individualized to the pathogen and patient evolution. For specific infections such as spinal tuberculosis or brucellar spondylitis, management adhered to established guideline recommendations. This protocol is based on our institutional guidelines, integrating published evidence, local stewardship principles, and clinical experience. Follow-up examinations should include periodic assessment of C-reactive protein (CRP), erythrocyte sedimentation rate (ESR), and procalcitonin(PCT) levels. Nutritional support was intensified, and sedative or analgesic medications were provided as needed. Patients were maintained on bed rest on the operative day. The drainage tube was removed 24 hours postoperatively. Patients were encouraged to mobilize early in the postoperative period while wearing a lumbar brace, undertaking progressive functional exercise under the supervision of a physical therapist that initially avoided flexion, rotation, and heavy lifting during training. Heavy physical labor and sports activities were prohibited for 1 months postoperatively.

### 2.5 Observation parameters and follow-up methods

Outcome measures included general surgical data, radiological parameters, and clinical outcomes. All radiographic parameters were independently measured by two spine surgeons, and inter-observer reliability was assessed using the intraclass correlation coefficient (ICC), with excellent consistency confirmed (ICC > 0.90). The average values of the two observers were used for analysis. Follow-up concluded in June 2024. Operative time, surgical level, intraoperative blood loss, hospital stay, and surgical complications were recorded for all patients. Visual Analog Scale (VAS) scores for pain and Oswestry Disability Index (ODI) scores were recorded preoperatively and at 1, 3, 6, and 12 months postoperatively were assess the alleviation of back pain and functional improvement. Neurological status and recovery were evaluated using the American Spinal Injury Association (ASIA) Impairment Scale. Patient symptoms and complications were monitored until clinical stabilization and complete resolution of symptoms were achieved.

**Figure 2:**
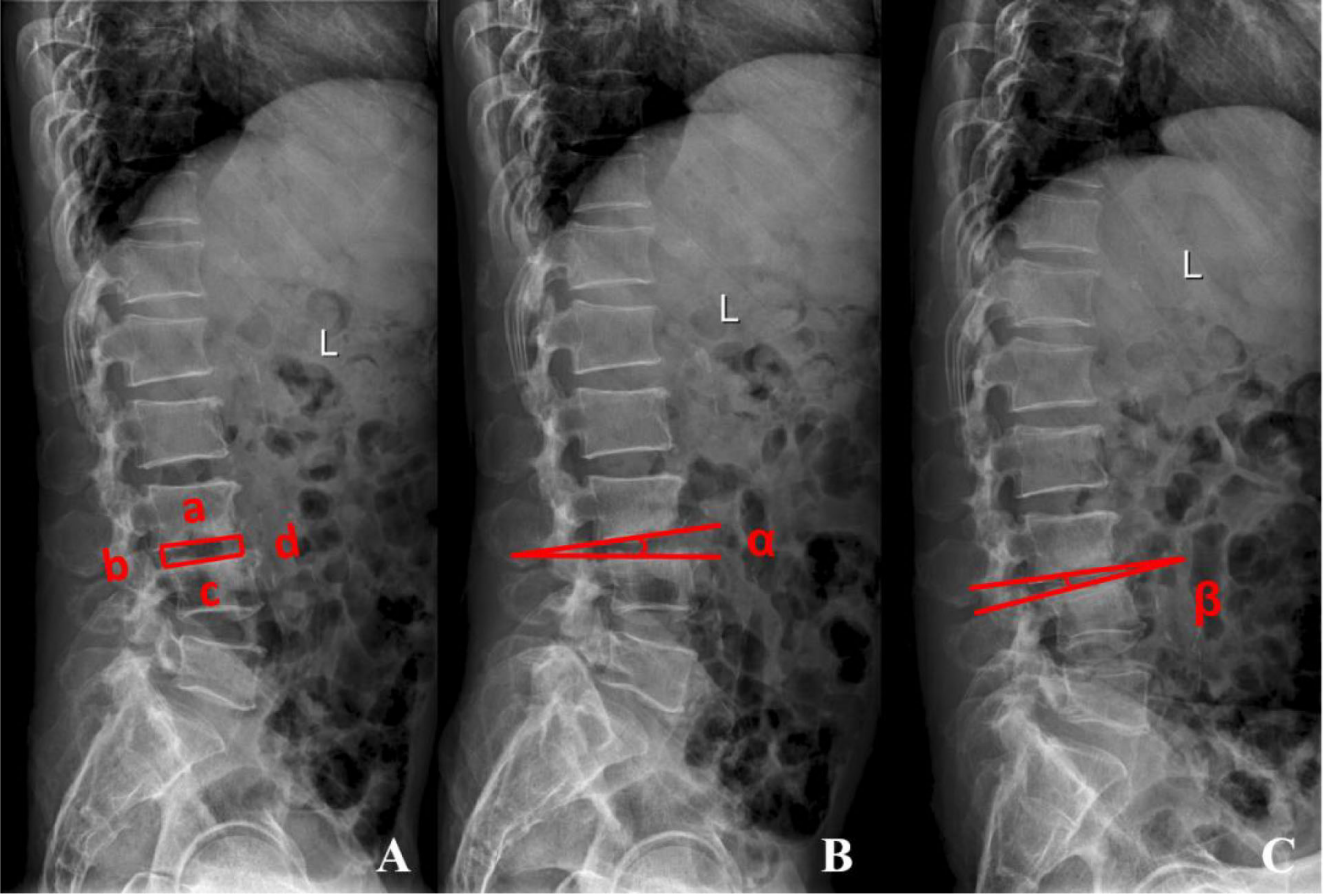
Radiographic index measurement methods: **(A)** Intervertebral Height Index (IHI)= (b + d)/(a + c)*100%. **(B, C)** Segmental Range of Motion(sROM)= |α - β| if angles in same direction; α + β if angles in opposite directions. ①Intervertebral Height Index: Measurement of affected intervertebral space height on preoperative and postoperative lateral radiographs. (a. Width of the inferior endplate of the upper vertebra; b. Height between posterior vertebral body lines; c. Width of the superior endplate of the lower vertebra; d. Height between anterior vertebral body lines.) ②Segmental Range of Motion: The intervertebral angle at the affected level is measured on preoperative and postoperative flexion and extension radiographs.

Postoperative imaging, performed on day 2, included anteroposterior and lateral lumbar radiographs as well as 3D reconstructed lumbar CT. Follow-up at 1, 3, 6, and 12 months included anteroposterior, lateral, and dynamic lumbar radiographs, in addition to lumbar CT and MRI to monitor lesion repair, intervertebral height, spinal stability, and the position of the cement beads. Preoperative and postoperative imaging data were collected (**Figure 2**). The Intervertebral Height Index (IHI) and segmental Range Of Motion (sROM) were measured and analyzed using ImageJ software (NIH, Bethesda, MD, USA).

### 2.6 Statistical analysis

Statistical analyses were conducted using SPSS software (version 31.0; SPSS Inc., Chicago, IL, USA). The normality of all quantitative data was assessed using the Shapiro-Wilk test. Normally distributed quantitative data are presented as mean ± standard deviation ( $\bar{\text{x}}\pm \text{s}$). Categorical data are expressed as numbers (percentages). For continuous variables, For continuous variables, repeated-measures analysis of variance (ANOVA) was applied, or the Friedman test when assumptions were not met. For ordinal variables such as ASIA grades, the Friedman test was used, followed by *post-hoc* pairwise comparisons with Bonferroni correction for all tests. A P-value< 0.05 was considered statistically significant.

## 3 Results

### 3.1 Demographics and surgical outcomes

Twelve patients were included in this study between March 2021 and June 2024. Baseline demographic characteristics are summarized in **Table 1**. All procedures were completed successfully, resulting in the effective resolution of lumbar spinal infections. The mean operative time was 85.58 ± 13.15 minutes, with an estimated blood loss of 51.83 ± 11.50 mL and a mean hospital stay of 6.50 ± 1.45 days. The mean follow-up time was 16.50 ± 4.20 months. Among the 12 patients, the specific diagnoses included pyogenic spondylodiscitis (8 cases), brucellar spondylitis (1 case), postoperative lumbar infection (2 cases), and spinal tuberculosis (1 case). Low back pain was present in 10 patients, and 5 had radicular symptoms. Additionally, 4 patients had varying degrees of fever (37.1 °C - 39 °C). Blood cultures were performed for those with temperatures exceeding 38.5 °C. Pathogen identification yielded the following results: Staphylococcus aureus (4 cases, 33.3%), Escherichia coli (2 cases, 16.7%), Staphylococcus epidermidis (2 cases, 16.7%), Mycobacterium tuberculosis (1 case, 8.3%), Streptococcus anginosus (1 case, 8.3%), and Brucella (1 case, 8.3%). No pathogen was detected in one case. Imaging findings indicated involvement at the following spinal segments: L2-3 (1 case, 8.3%), L3-4 (4 cases, 33.3%), L4-5 (5 cases, 41.7%), L5-S1 (2 cases, 16.7%). Significant vertebral bone destruction was observed in 10 cases, intraspinal epidural abscess formation in 4 cases. No complications occurred in the cohort, such as infection recurrence, cement bead migration, allergic reactions, cauda equina injury, cerebrospinal fluid leakage, or pars interarticularis fracture.

**Table 1:** General information of 12 patients.

Case	Age/Sex	Detailed diagnosis	Pathogenic bacteria	Lesion segment	Fever	ASIA
1	69/M	*Suppurative* sp*ondylitis*	*Staphylococcus aureus*	L3-4	Present	C
2	61/F	*Suppurative* sp*ondylitis*	*Staphylococcus aureus*	L3-4	Present	C
3	56/M	*Suppurative* sp*ondylitis*	*Staphylococcus aureus*	L2-3	Absent	C
4	62/M	*Suppurative* sp*ondylitis*	*Escherichia coli*	L5-S1	Absent	D
5	68/F	*Suppurative* sp*ondylitis*	*Negative*	L5-S1	Absent	D
6	73/F	*Postoperative infection*	*staphylococcus epidermidis*	L4-5	Absent	C
7	59/M	*Brucellar* sp*ondylitis*	*Brucella*	L3-4	Present	D
8	69/F	*Postoperative infection*	*staphylococcus epidermidis*	L4-5	Absent	C
9	63/M	*Spinal tuberculosis*	*Mycobacterium tuberculosis*	L4-5	Absent	D
10	59/F	*Suppurative* sp*ondylitis*	*Escherichia coli*	L4-5	Absent	E
11	66/M	*Suppurative* sp*ondylitis*	*Streptococcus anginosus*	L4-5	Present	C
12	67/M	*Suppurative* sp*ondylitis*	*Staphylococcus aureus*	L4-5	Absent	D

### 3.2 Clinical efficacy evaluation

Over time, patients experienced gradual resolution of recurrent fever, back pain, and lower limb neurological symptoms, accompanied by successful control and eventual cure of the infection. All surgical incisions healed primarily without deep infection or sinus tract formation. By the final follow-up, there were no cases of clinically or laboratory-confirmed infection recurrence. VAS and ODI scores at all postoperative follow-up intervals demonstrated significant improvement compared to preoperative values (P< 0.05). Postoperative inflammatory markers (WBC, ESR, CRP, PCT) decreased significantlyand returned to normal ranges compared to preoperative levels (P< 0.05). ASIA grades at postoperative and final follow-up also showed significant improvement compared to preoperative grades (P< 0.05) (**Tables 2**, **3**). According to the Fischgrund criteria, clinical outcomes were rated as excellent in 6 cases, good in 4 cases, and fair in 2 cases, yielding an overall excellent/good rate of 83%.

**Table 2:** Comparison of clinical and radiographic outcomes before and after surgery (n=12).

Parameter	Preoperative	Immediately postoperative	Final follow-up	*F* value	*P* value (Overall)	*P* value (*Post-hoc*, Bonferroni-adjusted)		
						Pre vs. Imm	Pre vs. final	Imm vs. final
Back pain VAS	6.50 ± 1.17	4.25 ± 0.97	2.50 ± 1.72	84.788	<0.05	<0.05	<0.05	<0.05
ODI	67.42 ± 2.27	33.33 ± 2.96	19.17 ± 3.76	787.610	<0.05	<0.05	<0.05	<0.05
WBC (*10^9/L)	13.47 ± 4.12	9.32 ± 1.13	8.17 ± 0.85	14.735	<0.05	<0.05	<0.05	<0.05
ESR (mm/h)	59.33 ± 16.04	28.58 ± 9.60	12.33 ± 4.42	55.570	<0.05	<0.05	<0.05	<0.05
CRP (mg/L)	71.63 ± 25.56	22.29 ± 6.26	3.42 ± 1.86	64.164	<0.05	<0.05	<0.05	<0.05
PCT (ng/ml)	1.59 ± 1.06	0.37 ± 0.37	0.04 ± 0.02	18.984	<0.05	<0.05	<0.05	>0.05
IHI	0.19 ± 0.04	0.24 ± 0.03	0.23 ± 0.02	7.509	<0.05	<0.05	<0.05	>0.05
sROM (°)	8.02 ± 1.46	8.09 ± 1.32	2.85 ± 1.10	64.060	<0.05	>0.05	<0.05	<0.05

Data are presented as mean ± standard deviation; *p* value (Overall) was determined by Repeated-Measures ANOVA (or Friedman Test if non-normal); *Post-hoc* pairwise comparisons were performed using Wilcoxon signed-rank test with Bonferroni correction. IHI, Intervertebral Height Index; sROM, sagital Range Of Motion; Pre, Preoperative; Imm, Immediately Postoperative; Final, Final Follow-up.

**Table 3:** Neurological outcomes according to ASIA impairment scale (n=12).

Assessment time point	ASIA grade, n(%)			Median grade (IQR)	*P* value (Overall)	Pairwise comparison	*Z* value	*P* (*Post-hoc*, Bonferroni-adjusted)
	C	D	E					
Preoperative	6 (50%)	5 (41.7%)	1 (8.3%)	C (C to E)	<0.05	Pre vs. Final	-2.919	<0.05
Immediately Postoperative	2 (16.7%)	6 (50%)	4 (33.3%)	D (C to E)		Pre vs. Imm	-1.897	<0.05
Final Follow-up	0	4 (33.3%)	8 (66.7%)	E (D to E)		Imm vs. Final	-2.111	0.083

Data are presented as mean ± standard deviation; *p* value (Overall) was determined by Friedman Test; *Post-hoc* pairwise comparisons were performed using Wilcoxon signed-rank test with Bonferroni correction; Pre, Preoperative; Imm, Immediately Postoperative; Final, Final Follow-up.

### 3.3 Radiographic evaluation

dMRI at 1 month postoperatively showed significantly reduced inflammatory signals in the intervertebral space and surrounding vertebrae compared to preoperative imaging in all patients, indicating effective local infection control. By the final follow-up, X-rays and 3D CT showed well-maintained spinal physiological curvature. Signs of repair were evident in the previously infected endplates and vertebral bones, characterized by reduced bone erosion, marginal sclerosis, and new bone formation, with no evidence of progressive bony destruction or vertebral collapse. Notably, 75% (9/12) of patients developed mature bridging osteophytes along the anterior margins of the affected segments, achieving bony stability in the anterior column. Flexion-extension radiographs obtained at the final follow-up showed a significantly reduced segmental range of motion compared to preoperative measurements, indicating spontaneous interbody fusion and satisfactory lumbar stability (P< 0.05) (**Figure 2**; **Table 2**). Immediate postoperative X-rays confirmed satisfactory positioning of the antibiotic cement beads in all cases, with all beads confined to the intervertebral space and debrided areas. The beads were evenly distributed without protrusion into the spinal canal or neural foramina, and no signs of neural compression were observed. To ensure sample homogeneity for intervertebral height measurement, The IHI was introduced. The IHI was well-maintained immediately postoperatively (increasing from 0.19 ± 0.04 to 0.24 ± 0.03). Although a slight decrease was observed by the final follow-up (0.23 ± 0.02), the height maintenance rate remained at 80% (**Figure 2**; **Table 2**).

## 4 Discussion

**Figure 3:**
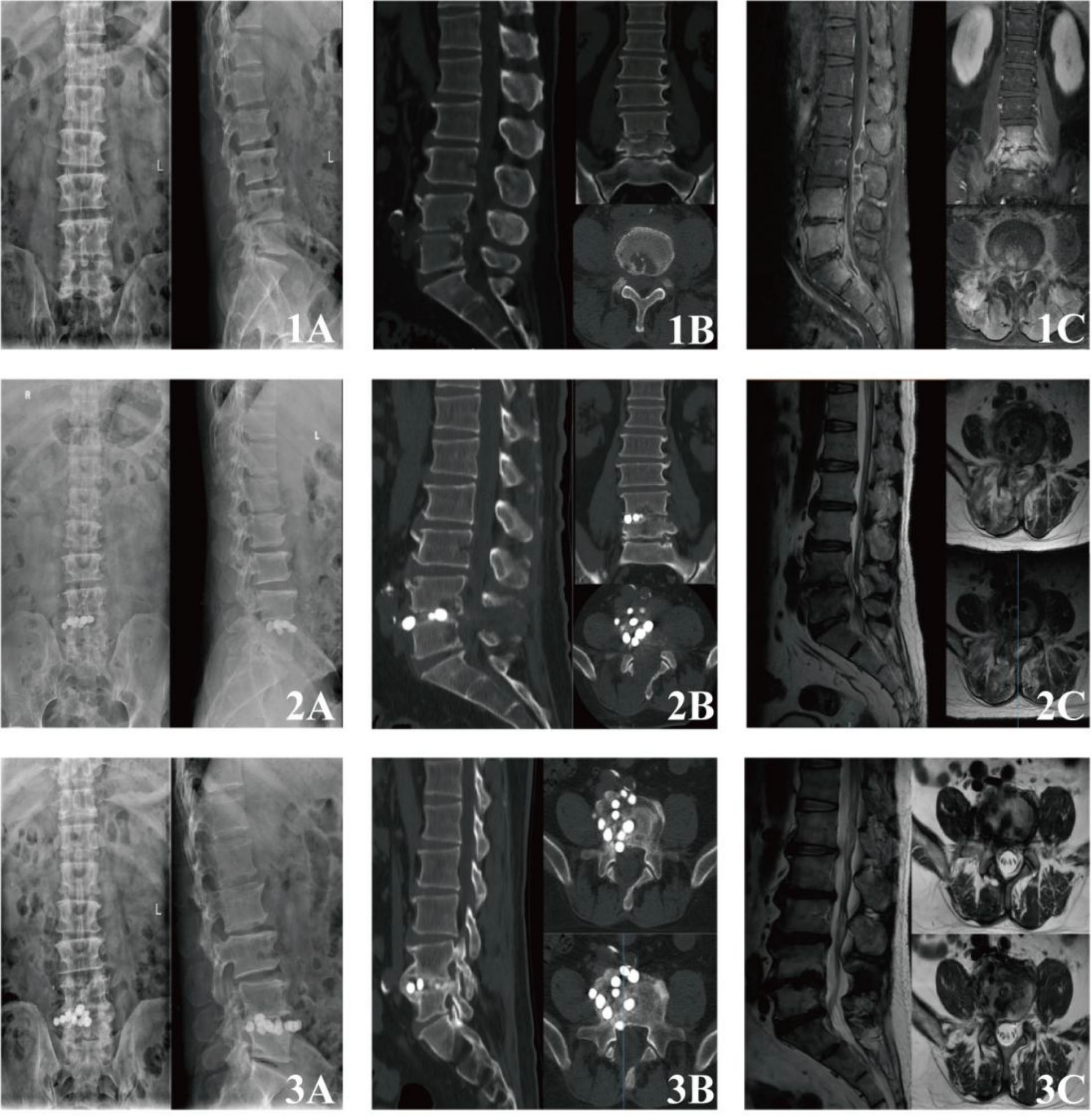
Preoperative and postoperative radiological imaging of typical cases: **(1A-1C)** Preoperative imaging. Radiography and CT demonstrate loss of disc height at L4–5, accompanied by endplate erosions and osteolytic destruction of the inferior endplate of L4 and superior endplate of L5. Contrast-enhanced MRI reveals diffuse enhancement of the L4 and L5 vertebral bodies, along with the posterosuperior portions of S1 and S2. The L4–5 disc shows irregular linear enhancement. An enhancing fluid collection (abscess) is observed within the spinal canal, extending cranially to the L2–3 level. **(2A-2C)** Immediate postoperative imaging. Radiography and CT confirm adequate positioning of radiopaque bone cement beads within the intervertebral space, which maintain distraction and restore segmental height. No significant bead fragmentation or migration is observed. MRI demonstrates marked regression of the pre-existing paraspinal and epidural abscesses. Bone marrow edema in the adjacent vertebrae is significantly reduced on T2-weighted sequences. The beads themselves appear as signal voids on all sequences. **(3A-3C)** Imaging at final follow-up. Radiography and CT show well-maintained vertebral and intervertebral height. Signs of osseous repair are evident at the previously infected sites, characterized by resolution of erosions with marginal sclerosis and new bone formation. There is no progressive bony destruction or vertebral collapse. Bone osteophytes have formed from the vertebral endplates, bridging the intervertebral space to form continuous bony bridges, indicating stable anterior column fusion. MRI confirms complete resolution of the previous vertebral bone marrow edema and paraspinal/epidural abscesses. The cement beads are encapsulated within a well-defined fibrous capsule.

This preliminary study suggests that UBE-assisted precise debridement combined with intervertebral implantation of ALCBs may represent a safe, effective, and feasible minimally invasive strategy for selected patients with lumbar spinal infections. A typical case is shown in **Figure 3**.

### 4.1 Value of intervertebral implantation of ALCBs

ALCBs could offer considerable advantages from both pharmacological and biomechanical perspectives. Pharmacologically, the primary value of ALCBs lies in their ability to deliver an ultra-high local concentrations of antibiotics directly at the infection site (Madadi and Sohn, 2024). Systemic intravenous administration is limited by the blood-disc barrier, making it difficult to achieve effective therapeutic concentrations in the poorly vascularized disc and surrounding necrotic tissue. In contrast, ALCBs can sustainably release antibiotic concentrations tens to hundreds of times higher than systemic administration, which may enable a more thorough elimination of bacteria within biofilms (Sweet et al., 2011). This property could make them particularly valuable for managing infections caused by drug-resistant bacteria such as *Methicillin-resistant Staphylococcus aureus* and *Pseudomonas aeruginosa* (Sweet et al., 2011; Dusane et al., 2017; Schwarz et al., 2021). Moreover, this localized release mechanism may effectively reduce the risk of hepatorenal toxicity associated with high-dose intravenous antibiotics and may offer a safer alternative for patients with comorbidities such as renal impairment (Dedeogullari et al., 2023; Gao et al., 2023). Notably, the antibiotic loaded into the bone cement beads was selected based on the specific etiology: vancomycin was used for patients with pyogenic spondylitis, while streptomycin was chosen for those with spinal tuberculosis or Brucella spondylitis. From a biomechanical and structural perspective, ALCBs also offer considerable benefits. Following debridement, the residual intervertebral space is prone to collapse and progressive kyphotic deformity. ALCBs can serve as a mechanical spacer by filling this cavity, providing what may be immediate structural support to help maintain intervertebral height and spinal alignment. This is likely to establish a favorable environment for spontaneous interbody fusion (Parker et al., 2004; Masuda et al., 2017; Tang et al., 2022). Compared to simple drainage or irrigation, this spacer effect may also reduce dead space formation, thereby lowering the risk of hematoma accumulation and recurrent infection (Glassman et al., 1996; Viswanathan et al., 2022).

### 4.2 Advantages of UBE technology in this combined technique

UBE technology demonstrates unique advantages in this combined procedure, extending far beyond a mere substitute for single-portal endoscopy. The primary advantage of this technique lies in the separation of visual and operational channels, which provides exceptional flexibility in instrumentation. By employing independent portals, the surgeon gains the freedom to use standard tools such as pituitary rongeurs, curettes, and osteotomes, an operative maneuverability that closely mirrors that of open surgery. This facilitates efficient removal of tightly adherent necrotic disc material and sequestra via shaving, scraping, and biting, thereby enabling thorough debridement of infected foci (Ju and Lee, 2023; He et al., 2024; Van Isseldyk et al., 2024). Simultaneously, the continuous irrigation system in UBE may help create a favorable hydrodynamic environment, which can facilitate the clearance of intraoperative bleeding, purulent secretions, and tissue debris. This is thought to help maintain a clearer surgical field, potentially aiding in the accurate distinction between infected granulation tissue and normal anatomy or critical neurovascular structures, thereby contributing to improved surgical safety and debridement completeness (Chu et al., 2025). Moreover, UBE technology allows for decompression and debridement of the contralateral spinal canal and intervertebral space lesions via a unilateral approach by flexibly adjustment of the endoscope angle and working channel. This technique avoids the additional trauma associated with traditional bilateral open surgery or uniportal coaxial endoscopy (He et al., 2024; Tang et al., 2024). Furthermore, the spacious working channel may greatly facilitate the implantation process. This design allows for the pre-formed antibiotic cement beads to be placed with accuracy and relative completeness into the prepared intervertebral space, often without requiring extended incisions or significantly risking bead displacement, which likely enhances the overall reliability and smoothness of the procedural execution.

A learning curve for the combined UBE and ALCB implantation technique must be recognized, as its success is inherently linked to procedural skill in endoscopy and infection management. All cases were performed by a single, high-volume surgeon who performs approximately 300 UBE procedures annually and has dedicated experience in managing complex spinal infections, which may have contributed to outcome uniformity. However, the magnitude of this learning curve was not quantified in our retrospective design. Thus, the influence of progressive surgical mastery on the reproducibility of our findings remains to be elucidated.

### 4.3 Selection of indications

This minimally invasive combined technique is not universally applicable to all types of lumbar spinal infections, its successful implementation strictly depends on appropriate patient selection. The ideal candidates are patients with pyogenic spondylodiscitis caused by identified pathogens, such as monomicrobial infections with Staphylococcus aureus or Streptococcus, and which are in the early to middle stages radiographically (Modic I-III). In these cases, lesions should primarily present with disc and subchondral bone marrow edema along with endplate erosion, without evidence of extensive bone destruction, vertebral compression fractures, or severe kyphosis. Additionally, there should be no significant mechanical spinal instability or neurological impairment requiring emergency open surgery (Modic et al., 1988; Gentile et al., 2019; Abreu et al., 2022). For specific infections such as spinal tuberculosis and brucellar spondylitis, definitive cure depends upon prolonged, pathogen-directed systemic antimicrobial therapy, which all corresponding patients in the present series completed per established guidelines. (**Supplementary Figures 1**, **2**) In carefully selected cases, including those with localized granulomatous spondylitis, early postoperative deep infection where existing instrumentation remains stable, and elderly patients with comorbidities precluding open surgery, this combined technique may be considered a strictly adjunctive procedure following comprehensive evaluation (Khanna and Sabharwal, 2019; Abulizi et al., 2021; Tai et al., 2024). Its principal surgical objectives are to obtain tissue for definitive microbiological diagnosis and drug sensitivity testing, to perform targeted debridement of the infectious nidus, and to achieve sufficient neural decompression to manage progressive neurological deterioration. Conversely, patients with advanced infection accompanied by severe structural destruction, spinal instability, or progressive kyphosis, those with large paravertebral or psoas abscesses requiring extensive drainage, infections involving three or more segments, cases with unidentified pathogens or multidrug-resistant bacteria requiring extensive debridement, and those with recurrence after previous conservative or minimally invasive treatment failure should be considered contraindications. Traditional open surgery is recommended for more radical management in these cases (Akgul et al., 2022; Kramer et al., 2024; Kramer et al., 2025).

### 4.4 Limitations and future prospects

Several limitations of this study should be acknowledged. Firstly, this was a single-center retrospective study with a limited sample size and lacks a control group treated with alternative surgical techniques, which may introduce selection bias and limit the generalizability of the results. Secondly, while the mean follow-up of 16.5 months allowed assessment of short- to mid-term infection control and early fusion, it may be insufficient to evaluate long-term outcomes such as late infection recurrence, durability of spontaneous interbody fusion, or potential late-onset complications related to cement retention. Furthermore, the concomitant use of postoperative systemic antibiotic therapy in all patients represents a potential confounding factor, making it difficult to isolate the individual contribution of the locally implanted ALCBs to infection control.

Based on these limitations, future research should focus on the following directions: conducting multicenter prospective randomized controlled trials to compare the efficacy and safety of this minimally invasive technique with other surgical and medical treatments; eveloping biodegradable antibiotic-eluting carriers to overcome the non-degradable nature of PMMA and optimize drug-release kinetics; exploring personalized antibiotic selection based on advanced pathogen profiling and local susceptibility testing; and exploring local combination antibiotic therapy, resistance prevention strategies, and the application of this technique in cervical and thoracic spinal infections to broaden its clinical applicability.

## 5 Conclusion

In summary, within the limitations of this small retrospective series, the combined UBE and ALCB technique appears to be a safe and feasible minimally invasive approach for selected patients with lumbar spinal infections. It integrates the advantages of enhanced visualization, thorough debridement, and targeted local antibiotic delivery, representing a promising therapeutic direction. These early results are encouraging but underscore the need for prospective, controlled studies to further validate its efficacy and safety profile.

## Data Availability

The raw data supporting the conclusions of this article will be made available by the authors, without undue reservation.
