# Supplementary material for: Unilateral biportal endoscopic debridement with antibiotic-loaded cement beads implantation for lumbar spinal infection: a preliminary report on feasibility and early clinical outcomes
Source: Front Cell Infect Microbiol. 2026 Mar 18;16:1752848. doi: 10.3389/fcimb.2026.1752848 (PMC13039001; doi:10.3389/fcimb.2026.1752848)
Supplement: Supplementary file 1 [file Presentation1.pptx]

## Slide 1
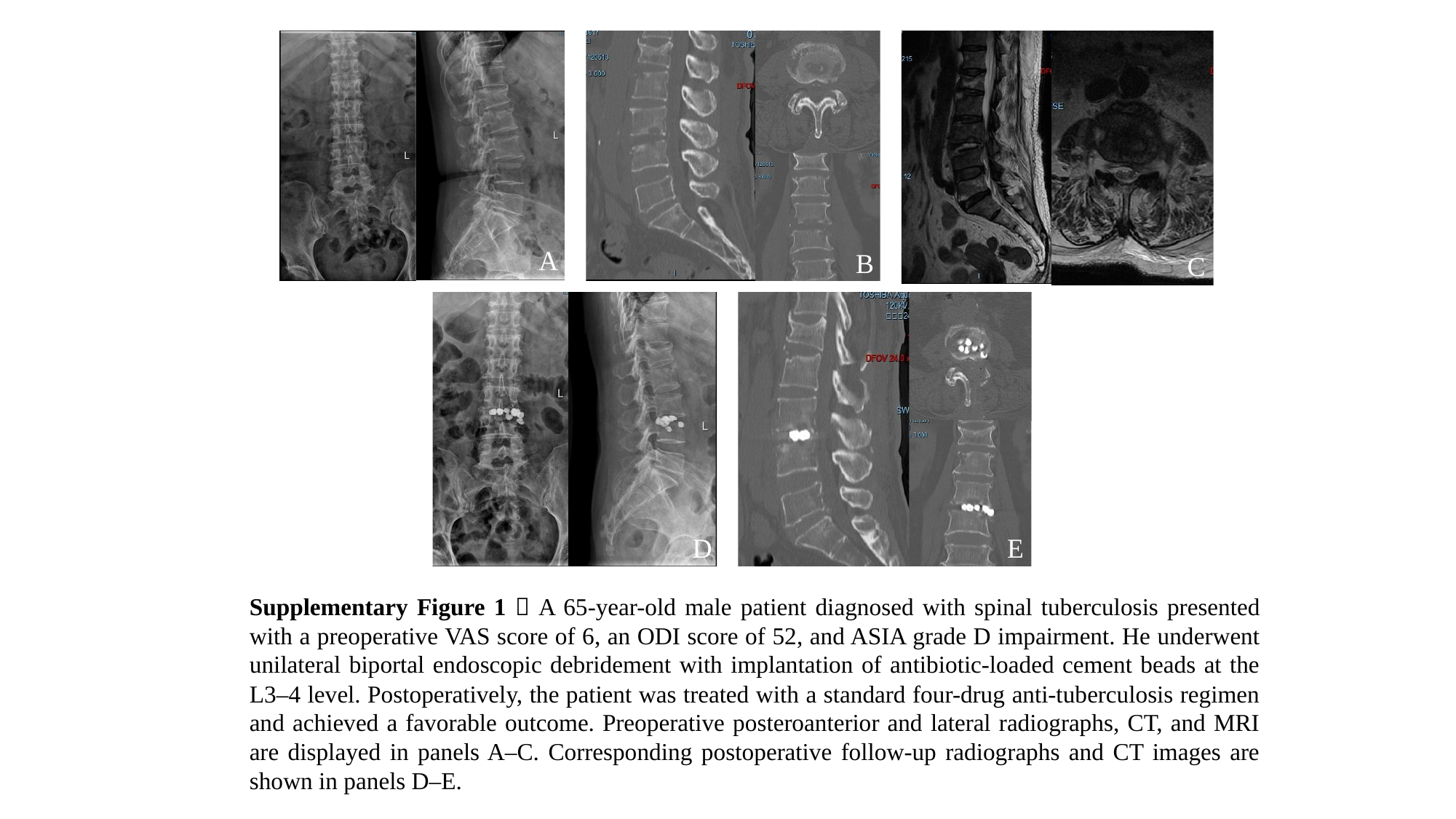

A
B
C
D
E
Supplementary Figure 1：A 65-year-old male patient diagnosed with spinal tuberculosis presented with a preoperative VAS score of 6, an ODI score of 52, and ASIA grade D impairment. He underwent unilateral biportal endoscopic debridement with implantation of antibiotic-loaded cement beads at the L3–4 level. Postoperatively, the patient was treated with a standard four-drug anti-tuberculosis regimen and achieved a favorable outcome. Preoperative posteroanterior and lateral radiographs, CT, and MRI are displayed in panels A–C. Corresponding postoperative follow-up radiographs and CT images are shown in panels D–E.

## Slide 2
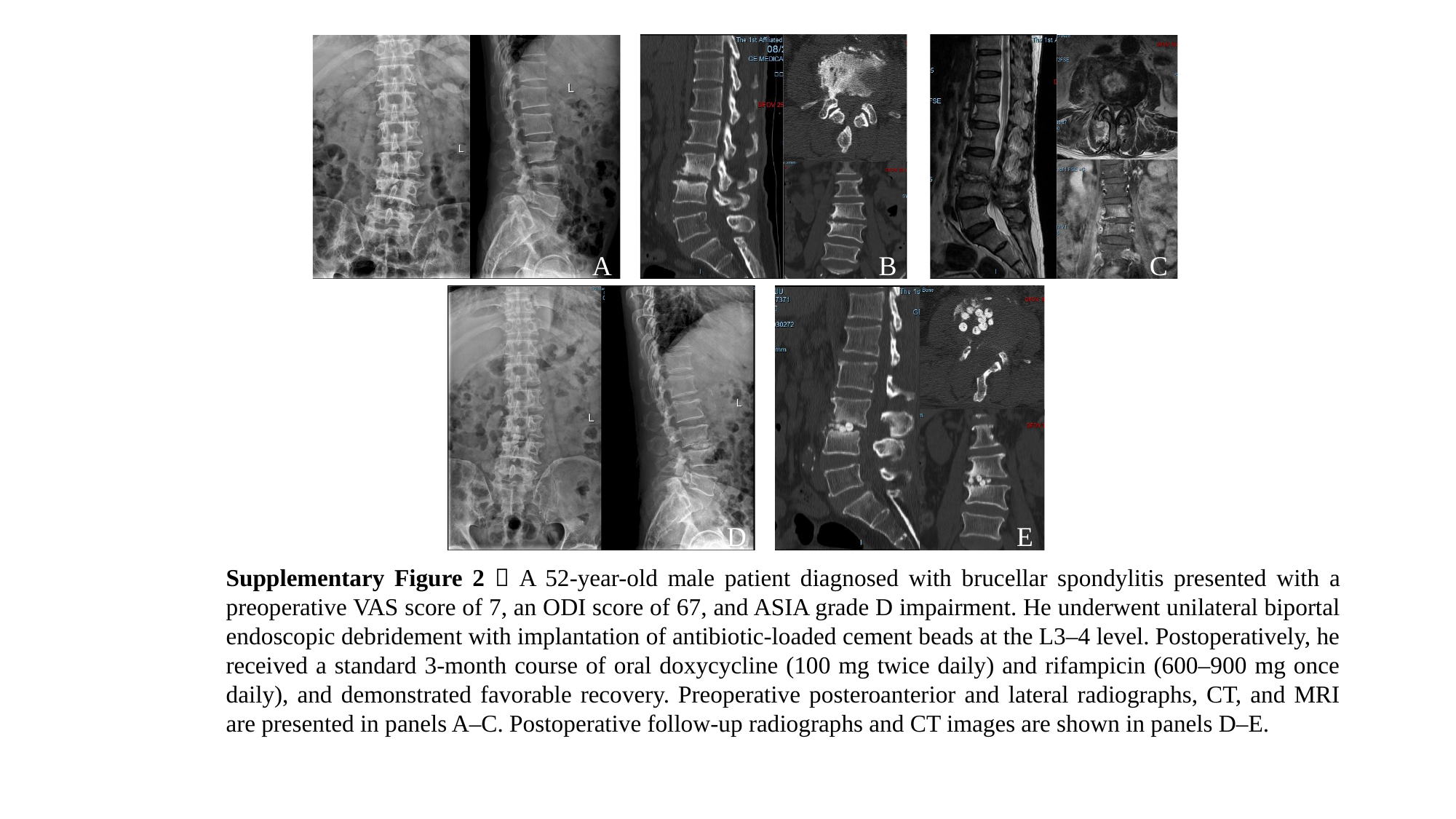

A
B
C
D
E
Supplementary Figure 2：A 52-year-old male patient diagnosed with brucellar spondylitis presented with a preoperative VAS score of 7, an ODI score of 67, and ASIA grade D impairment. He underwent unilateral biportal endoscopic debridement with implantation of antibiotic-loaded cement beads at the L3–4 level. Postoperatively, he received a standard 3-month course of oral doxycycline (100 mg twice daily) and rifampicin (600–900 mg once daily), and demonstrated favorable recovery. Preoperative posteroanterior and lateral radiographs, CT, and MRI are presented in panels A–C. Postoperative follow‑up radiographs and CT images are shown in panels D–E.
